# Cadherin-13 Deficiency Increases Dorsal Raphe 5-HT Neuron Density and Prefrontal Cortex Innervation in the Mouse Brain

**DOI:** 10.3389/fncel.2017.00307

**Published:** 2017-09-26

**Authors:** Andrea Forero, Olga Rivero, Sina Wäldchen, Hsing-Ping Ku, Dominik P. Kiser, Yvonne Gärtner, Laura S. Pennington, Jonas Waider, Patricia Gaspar, Charline Jansch, Frank Edenhofer, Thérèse J. Resink, Robert Blum, Markus Sauer, Klaus-Peter Lesch

**Affiliations:** ^1^Division of Molecular Psychiatry, Center of Mental Health, University of Würzburg, Würzburg, Germany; ^2^Department of Biotechnology and Biophysics, Biocenter, University of Würzburg, Würzburg, Germany; ^3^Institut du Fer á Moulin, Institut National de la Santé et de la Recherche Médicale (INSERM), UMR-S839, Paris, France; ^4^Department of Genomics, Stem Cell Biology and Regenerative Medicine, Institute of Molecular Biology and Center for Molecular Biosciences Innsbruck (CMBI), Leopold-Franzens-University Innsbruck, Innsbruck, Austria; ^5^Stem Cell Biology and Regenerative Medicine Group, Institute of Anatomy and Cell Biology, Julius-Maximilians-University of Würzburg, Würzburg, Germany; ^6^Laboratory for Signal Transduction, Department of Biomedicine, University Hospital Basel, University of Basel, Basel, Switzerland; ^7^Department of Clinical Neurobiology, University of Würzburg, Würzburg, Germany; ^8^Laboratory of Psychiatric Neurobiology, Institute of Molecular Medicine, I. M. Sechenov First Moscow State Medical University, Moscow, Russia; ^9^Department of Translational Neuroscience, School of Mental Health and Neuroscience, Maastricht University, Maastricht, Netherlands

**Keywords:** serotonin, cadherin-13 (CDH13), T-cadherin, neurodevelopment, psychiatric disorders, radial glia, dorsal raphe, prefrontal cortex

## Abstract

**Background:** During early prenatal stages of brain development, serotonin (5-HT)-specific neurons migrate through somal translocation to form the raphe nuclei and subsequently begin to project to their target regions. The rostral cluster of cells, comprising the median and dorsal raphe (DR), innervates anterior regions of the brain, including the prefrontal cortex. Differential analysis of the mouse 5-HT system transcriptome identified enrichment of cell adhesion molecules in 5-HT neurons of the DR. One of these molecules, cadherin-13 (Cdh13) has been shown to play a role in cell migration, axon pathfinding, and synaptogenesis. This study aimed to investigate the contribution of Cdh13 to the development of the murine brain 5-HT system.

**Methods:** For detection of Cdh13 and components of the 5-HT system at different embryonic developmental stages of the mouse brain, we employed immunofluorescence protocols and imaging techniques, including epifluorescence, confocal and structured illumination microscopy. The consequence of *CDH13* loss-of-function mutations on brain 5-HT system development was explored in a mouse model of Cdh13 deficiency.

**Results:** Our data show that in murine embryonic brain Cdh13 is strongly expressed on 5-HT specific neurons of the DR and in radial glial cells (RGCs), which are critically involved in regulation of neuronal migration. We observed that 5-HT neurons are intertwined with these RGCs, suggesting that these neurons undergo RGC-guided migration. Cdh13 is present at points of intersection between these two cell types. Compared to wildtype controls, Cdh13-deficient mice display increased cell densities in the DR at embryonic stages E13.5, E17.5, and adulthood, and higher serotonergic innervation of the prefrontal cortex at E17.5.

**Conclusion:** Our findings provide evidence for a role of CDH13 in the development of the serotonergic system in early embryonic stages. Specifically, we indicate that Cdh13 deficiency affects the cell density of the developing DR and the posterior innervation of the prefrontal cortex (PFC), and therefore might be involved in the migration, axonal outgrowth and terminal target finding of DR 5-HT neurons. Dysregulation of CDH13 expression may thus contribute to alterations in this system of neurotransmission, impacting cognitive function, which is frequently impaired in neurodevelopmental disorders including attention-deficit/hyperactivity and autism spectrum disorders.

## Introduction

The factors involved in the developmental program that tightly regulates neuronal migration and circuit formation of the brain serotonin (5-HT) system remain largely unknown. The development of this complex system commences early in prenatal stages, with 5-HT initially being supplemented from placental source (Wallace and Lauder, 1983; Bonnin and Levitt, 2011). It begins with the migration of 5-HT specific neurons for the arrangement of nine anatomically distinct groups of cells known as the raphe nuclei. These nuclei are subdivided into two main clusters: a caudal cluster in the medulla (B1–B5), and a rostral cluster in the pons (B6–B9). The most rostral cluster, composed of the median raphe (MR) and dorsal raphe (DR), is responsible for the innervation of anterior regions of the brain (Alonso et al., 2013). The identity of the 5-HT neuron population comprising the rostral cluster is determined by a transcription code that shows some variation along dorsoventral and anteroposterior axes (Ye et al., 1998; Gaspar et al., 2003; Kiyasova and Gaspar, 2011).

Early serotonin signaling plays a crucial role in CNS functions (Daubert and Condron, 2010), and it has been shown that both increased and decreased serotonin neurotransmission at different periods of embryonic and postnatal life compromises cortical development (Gaspar et al., 2003; Vitalis et al., 2007; Teissier et al., 2017). Similarly, dysfunction of 5-HT transmission has been implicated in neurodevelopmental disorders and subsequent psychiatric conditions in which social cognitive functions are compromised (Lesch and Waider, 2012). For instance, reduced serotonin levels were found in the frontal cortex of fetal Down syndrome (Whittle et al., 2007), and local developmental perturbations of 5-HT were identified in the brain of children with autism (Chugani et al., 1999). In addition, autistic-like symptoms were induced in rodents when decreasing (Boylan et al., 2007) or increasing (McNamara et al., 2008) brain 5-HT during development.

Calcium-dependent cell adhesion molecules, also known as cadherins, are mediators in cell migration and neural circuit formation during early stages of development (Redies, 1995; Halbleib and Nelson, 2006; Takeichi, 2007). A transcriptome analysis of specific neuronal subpopulations from mouse hindbrain, performed by combining intersectional fate mapping, cell sorting, and genome-wide RNA-sequencing, identified several cell adhesion molecules in rostral raphe nuclei, with the expression of cadherin-13 (*Cdh13*) being specifically restricted to 5-HT neurons of the DR (Okaty et al., 2015).

CDH13 (also known as T-cadherin), is an atypical cadherin that lacks both transmembrane and cytoplasmic domains, and is instead attached to the cell membrane through a glycosylphosphatidylinositol anchor (Ranscht and Dours-Zimmermann, 1991). CDH13 regulates cell migration, neurite outgrowth, axon guidance and target finding via low-adhesive homophilic and heterophilic interactions (Ranscht and Bronner-Fraser, 1991; Fredette and Ranscht, 1994; Fredette et al., 1996; Takeuchi et al., 2000; Bai et al., 2006; Ciatto et al., 2010; Hayano et al., 2014). Particularly, it acts as a negative regulator in the projection of motor neurons. Moreover, CDH13 is required for the development and proper functioning of glutamatergic and GABAergic synapses (Paradis et al., 2007; Rivero et al., 2015).

*CDH13* variation has been associated with neurodevelopmental and psychiatric disorders in numerous linkage, copy-number variant (CNVs), genome-wide association (GWAS), and whole-exome sequencing (WES) studies. Several studies have observed a reproducible association with attention-deficit/hyperactivity disorder (ADHD) (Lasky-Su et al., 2008; Lesch et al., 2008; Neale et al., 2008, 2010; Zhou et al., 2008; Lionel et al., 2011) and comorbid conditions, specifically substance use and dependence (Uhl et al., 2008a,b; Treutlein et al., 2009). Furthermore, common *CDH13* variants have been associated with cognitive functioning (e.g., performance in working memory tasks) in ADHD patients (Arias-Vasquez et al., 2011). Other studies relate CDH13 to depression (Sibille et al., 2009; Terracciano et al., 2010), bipolar disorder (Xu et al., 2014) and schizophrenia (Borglum et al., 2014). Rare *de novo* and inherited deletions (and less frequent duplications) at the *CDH13* locus have been linked to autism spectrum disorders (Sanders et al., 2011, 2015), indicating potential clinical relevance for loss-of-function mutations in *CDH13*. An association of *CDH13* SNPs with the personality trait of extraversion (Terracciano et al., 2010) and extremely violent behavior (Tiihonen et al., 2015) was also reported. However, the pathogenetic mechanisms by which *CDH13* variation influences behavior and the risk for neuropsychiatric disorders have not yet been clarified.

Given the role of CDH13 in cell migration, axon pathfinding, and synaptogenesis, the aim of this study was to characterize the expression pattern of Cdh13 during mouse brain development at different embryonic stages and to explore the relationship between Cdh13 and 5-HT system formation. Additionally, the consequence of CDH13 deficiency on brain 5-HT system development was investigated in a *Cdh13* knockout mouse, a model for loss-of function mutations at the *CDH13* locus presumed to cause neurodevelopmental disorders.

## Materials and methods

### Animals

All experimental procedures involving live animals were approved by the boards of the University of Würzburg and the Government of Lower Franconia and performed in accordance with the guidelines for animal care and use provided by the European Community. All experiments were carried out using a constitutive *Cdh13* knockout mouse line (*Cdh13*^−/−^, C57BI/6N genetic background) previously generated at the Division of Molecular Psychiatry, University of Würzburg (Rivero et al., 2015). Mice were housed in groups of 3–5 per cage at the facilities of the Center of Experimental Molecular Medicine, under a 12 h light/dark cycle with food and water *ad libitum*. For this study, *Cdh13*^−/−^ and *Cdh13*^+/+^ embryos were produced by crossing heterozygous animals (*Cdh13*^+/−^). Timed breedings were conducted over night and midday controls of plug positive animals were considered as embryonic day (E) 0.5.

### Embryo retrieval and tissue preparation

#### Embryos

Timed-pregnant dams were sacrificed through an overdose of isoflurane, and embryos were extracted at three different developmental stages: E13.5, E15.5, and E17.5. The brains from E15.5 and E17.5 embryos were dissected, while for stage E13.5 the complete head was processed. A small sample of the most caudal part of each embryo was taken for genotyping of the *Cdh13* locus (Rivero et al., 2015). Fixation of E13.5 heads as well as E15.5 and E17.5 brains was done by immersion in 4% paraformaldehyde (1xPBS; pH 7.5) at 4°C for 24 h, followed by cryoprotection in 10 and 20% sucrose solutions for 1 day each consecutively. The brains were frozen in isopentane cooled with dry ice and cryosectioned in coronal or sagittal 20 μm sections.

#### Adults

Adult mice were sacrificed at 2–3 months of age. This was done through an overdose of isoflurane followed by transcardial perfusion. Then the brains were dissected and kept in 4% paraformaldehyde (1xPBS; pH 7.5) overnight, and consecutively placed in 10 and 20% sucrose solutions for 1 day each for cryoprotection. The brains were then frozen in isopentane cooled with dry ice and cryosectioned in coronal or sagittal 20 μm sections.

### Indirect immunofluorescence

Brain sections were allowed to air dry for 30–45 min at room temperature and then washed in TBS and treated with citrate buffer, 10 mM, pH 6, at 80°C in a water bath for antigen retrieval. Sections were blocked with 10% normal horse serum and 0.2% Triton X-100 in TBS for 1 h, and then, incubated overnight at 4°C in a wet chamber with primary antibodies polyclonal goat anti-Cdh13 (1:200, R&D Systems, Minneapolis, USA, cat# AF3264), polyclonal rabbit anti-5-HT (1:500, Acris, Herford, Germany, cat# 20080), mouse anti-RC2 IgM monoclonal (1:60, Developmental Studies Hybridoma Bank, Iowa, USA, cat# AB531887), mouse anti-Nestin monoclonal (1:200, Santa Cruz Biotechnology, Dallas, USA, cat# SC-33677), rabbit anti-Serotonin (5-HT) transporter (1:500, Merck Millipore, cat# PC177L), rabbit anti-tryptophan hydroxylase 2 (TPH2) (1:1,000, self-produced; Gutknecht et al., 2008, 2009), and/or rabbit anti-OTX2 (1:500, Merck Millipore, cat# AB9566). The next day, sections were washed with TBS and incubated at room temperature with the corresponding secondary antibody, donkey anti-Goat IgG (H+L) Alexa Fluor 555, donkey anti-Mouse IgG (H+L) Alexa Fluor 488, donkey anti-Mouse IgM (H+L) Alexa Fluor 488, donkey anti-Rabbit IgG (H+L) Alexa Fluor 488, and/or donkey anti-Rabbit IgG (H+L) Alexa Fluor 647. Finally, 4′,6-Diamidin-2-phenylindol (DAPI) was applied as a nuclear counterstain to the sections, which were then embedded with Fluorogel as mounting medium (Electron Microscopy Sciences, Hatfield, USA).

### Imaging

Stained sections were imaged using one or more of the following microscopy techniques: (1) epifluorescence, (2) confocal microscopy, and/or (3) structured illumination microscopy (SIM).

#### Epifluorescence

Images were obtained using an Olympus motorized inverted system microscope IX81, an X-Cite fluorescence illuminator, and an XM10 camera. Pictures were taken at 10x, 20x(air), and/or 40x(oil) magnifications through the three exposure channels for Alexa Fluor 488, Cy3/Alexa Fluor 555, and DAPI. Images were then processed using software CellSense (Olympus, Leinfelden-Echterdingen, Germany), and corrected for contrast and brightness using ImageJ v2.0.0 (Schneider et al., 2012).

#### Confocal microscopy

Images were generated using a FluoView FV1000 confocal microscope (Olympus) with 20X UPlanSAPO, NA 0.75 (air) and 40X UPlanFLN, NA 1.30(oil) objectives. Stack images were taken by laser illumination at 561 nm (Alexa Fluor 555), 488 nm (Alexa Fluor 488), and 405 nm (DAPI). 12-bit raw images were processed with the imaging software Fluoview, version 4.1.a (Olympus).

#### Structured illumination microscopy (SIM)

Images were captured with a commercial inverted SIM microscope (Zeiss ELYRA, Oberkochen, Germany) using an oil-immersion objective (Plan-Apochromat 63 × /1.4 Oil Dic M27) (Gustafsson, 2000; Wegel et al., 2016). Excitation of the fluorophores was performed by laser illumination at 642 nm (Alexa Fluor 647), 561 nm (Alexa Fluor 555), 488 nm (Alexa Fluor 488), and 405 nm (DAPI) and fluorescence light was filtered by appropriate detection filters: LP 655 (Alexa Fluor 647), BP 570–620 + LP 750 (Alexa Fluor 555), BP 495–550 + LP 750 (Alexa Fluor 488), and BP 420–480 + LP 750 (DAPI). Images were recorded with five rotations and five phase steps of the illumination pattern. Recorded data were processed with the ZEN imaging software (Zeiss). They were processed under standard ELYRA settings of the manual mode, selecting the Raw Scale option to keep the original dynamic range and therefore ensure a reliable comparison of the actual sample and the control samples. Following the structured illumination processing, the four channels were aligned (ZEN imaging software).

### Three-dimensional reconstruction

For three-dimensional (3D) visualization of SIM images, z-stacks with intervals of 125 nm were recorded (usually ~50 slices). 3D reconstruction and animation of the processed SIM images were performed in Imaris (Bitplane, Zurich, Switzerland). The fluorescence signal was represented by surface visualization. Resulting 3D renderings were animated by rotation and zooming and exported as.avi files.

### Cell density in dorsal raphe and median raphe

Cell density measurements were carried out at E13.5, E17.5, and in the adult brain on ImageJ v2.0.0 by an observer unaware of genotype. At E13.5 the complete DR was imaged and then selected as the region of interest (ROI) in each image. Within this ROI, five areas of 50 × 50 pixels were then randomly selected. Using the Cell Counter plugin, the number of 5-HT immunoreactive (ir) cells in these areas (including the cells on the left and bottom borders of the areas) was counted. This was done through the entire stack of images, using DAPI as a counterstaining to count only cells with a focused nucleus and therefore avoid double counts. Then the average number of cells for each brain was calculated. At E17.5 and in the adult brain, sections at a distance of 120 μm were imaged, ~4–5 images per brain. In these images, the DR was selected as the ROI and then all the 5-HT-ir cells for E17.5 and TPH2-ir cells for adult brains were counted using the Cell Counter plugin, avoiding double counts through the use of DAPI. Finally, the average number of cells per brain was calculated.

### Area measurement of dorsal raphe

The area of the DR was measured using ImageJ v2.0.0. Six sections stained for 5-HT where the DR was clearly visible were selected per brain and the area of the group of 5-HT-ir cells was measured. Images where the DR extended out of the image were excluded.

### Serotonergic fiber number and density in prefrontal cortex

The area of the 5-HT-ir fibers at E17.5 and 5-Htt-ir fibers in adult brains was measured using ImageJ v2.0.0 as described previously (Gomez et al., 2007). At E17.5, six images of the prefrontal cortex were taken per brain. A threshold was set for each image to balance the signal-to-noise ratio by an observer unaware of genotype, so that the serotonergic fibers were clearly distinguishable from the background. Then, the images were transformed into binary images in which only immunostained elements were visible. A ROI of 100 × 100 pixels in the center of the intermediate zone (IZ) was set and the area of the fibers was calculated using the “Analyze Particles” option. In the adult brain, images of infralimbic (IL) and cingulate (CG) cortices from three to four sections at intervals of 120 μm were taken at 20x magnification. After backgrounds were subtracted and fibers were skeletonized, an evenly spaced square grid (70 × 70 μm^2^) was laid over the composite images. Finally, the area of the fibers within this ROI was calculated using the “Analyze Particles” option.

Additionally, in the adult brain prefrontal cortex, the number of 5-Htt immunopositive fibers intersecting three applied lines within selected areas was counted manually by plotting an intensity profile (Supplementary Figure 1). Fibers that touched the edge of the square were not counted. The results were presented as the number of fibers per μm^2^.

### Statistical analysis

Statistical analysis was performed using Prism, version 7.0a (GraphPad Software, La Jolla, CA, USA). The normality of the data sets was verified using the Kolmogorow-Smirnow test and the Shapiro-Wilk test. Once a normal distribution was confirmed, a two-tailed unpaired *t*-test was applied.

## Results

### Cdh13 follows a caudal to rostral progression in the developing mouse brain

**Figure 1:**
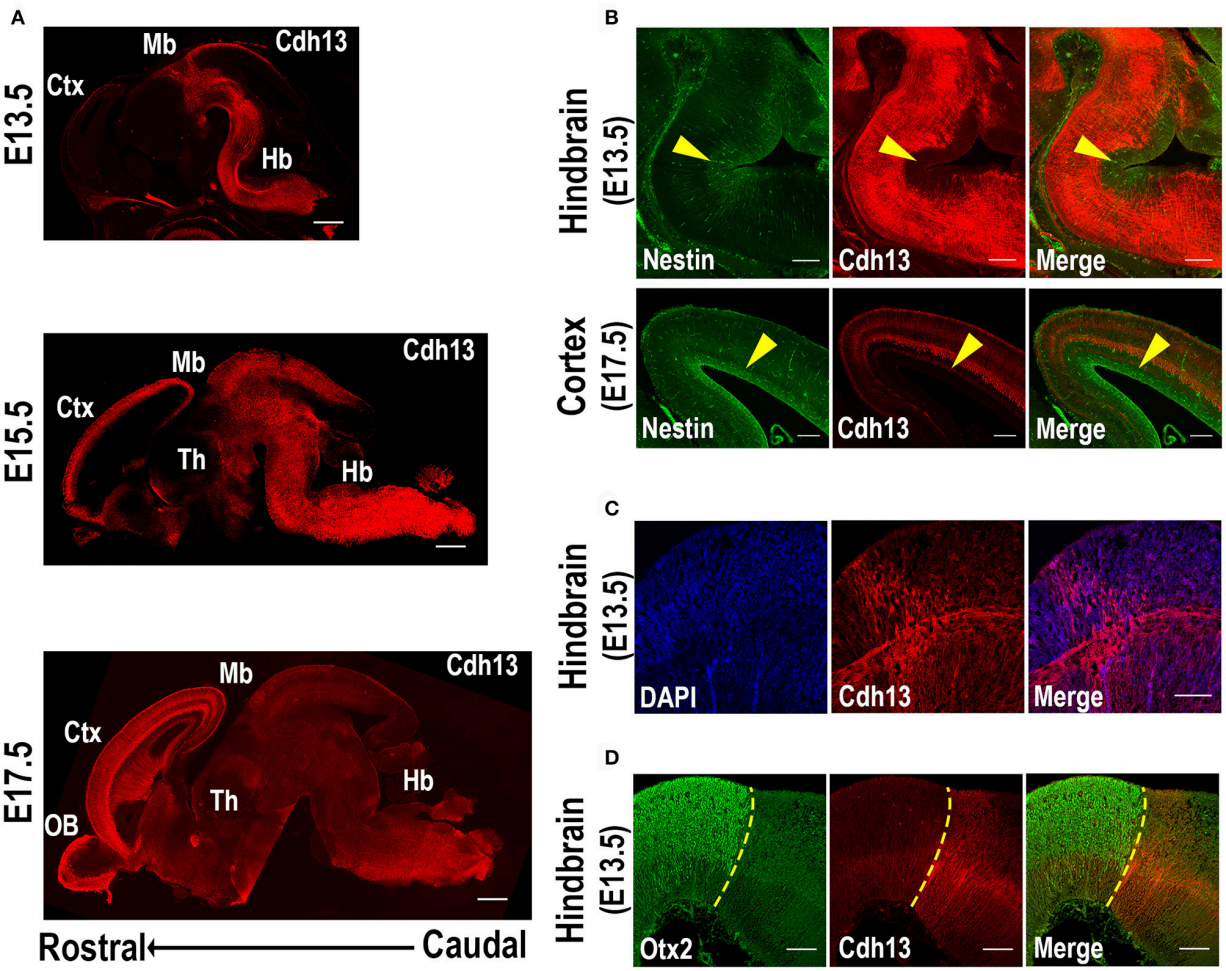
Cdh13 in embryonic mouse brain at selected developmental stages. **(A)** Cdh13 immunoreactivity is prominent in the hindbrain at E13.5, extends to various brain regions including the cortex at E15.5 and displays a layer-associated expression in the cortex at E17.5. **(B)** Cdh13 is not localized in regions immunoreactive for Nestin (yellow arrow: area positive for Nestin). **(C)** Cdh13 is found in the hindbrain at E13.5 in two groups of fibers that extend longitudinally and transversally **(D)** Cdh13 immunoreactivity in the hindbrain is juxtaposed to Otx2 midbrain staining, delimiting the MHB at E13.5 (yellow dotted line: MHB). Orientation: sagittal. Scale bars in **(A)** 500 μm, in **(B)** 200 μm, in **(C,D)** 100 μm. Ctx, cortex; Hb, hindbrain; Mb, midbrain; Th, thalamus; OB, olfactory bulb.

In order to determine the regional and cellular specificity of Cdh13 at different developmental stages and to identify variation in the cellular arrangement as the embryo developed, we conducted an analysis of the expression pattern, using the Allen Developing Mouse Brain Atlas (http://developingmouse.brain-map.org/) as a reference. The earliest developmental stage studied was E13.5 because at earlier embryonic days (E10.5 and E11.5) Cdh13 immunoreactivity is very low in the brain (Rivero et al., 2015). At stage E13.5, Cdh13 protein is limited to the hindbrain and midbrain, and almost completely absent from more anterior regions (Figure 1A, top). At the following stage E15.5, Cdh13 is evenly distributed throughout the different areas of the brain, spreading homogenously from the hindbrain to the telencephalon (Figure 1A, middle). At the latest stage E17.5, the expression pattern shifts, with the most intense cellular immunoreactivity being detected in the developing cortical layers (Figure 1A, bottom). Therefore, Cdh13 expression follows a caudal-to-rostral trajectory through development, showing an early detection in fibers originating from the hindbrain, which spread throughout the brain and finally concentrate in the cortex.

Along with its caudal-to-rostral progression, Cdh13 is restricted to the migratory path as it advances, with little to no expression in areas of neurogenesis. Specifically, Cdh13 was not detected in ventricular zones positive for Nestin, a marker of neural stem cells or progenitor cells (Lendahl et al., 1990). Instead, Cdh13-immunoreactive fibers delimit these regions of neurogenesis both in cortical areas as well as in the developing hindbrain (Figure 1B).

Cdh13-positive fibers in the hindbrain are also found to follow two distinct perpendicular directions in some regions at E13.5 (Figure 1C). More specifically, in rhombomere 1, adjacent to the midbrain-hindbrain boundary (MHB), one group of fibers extends longitudinally from caudal regions of the hindbrain and progresses into the midbrain, while another group projects transversally, extending from the ventricular zone of the hindbrain to the corresponding floorplate. Double-staining with Otx2, a protein that delimits the caudal boundary of the MHB (Li and Joyner, 2001), confirms that Cdh13-positive fibers extend transversally in the hindbrain until this midbrain boundary at E13.5 (Figure 1D).

**Figure 2:**
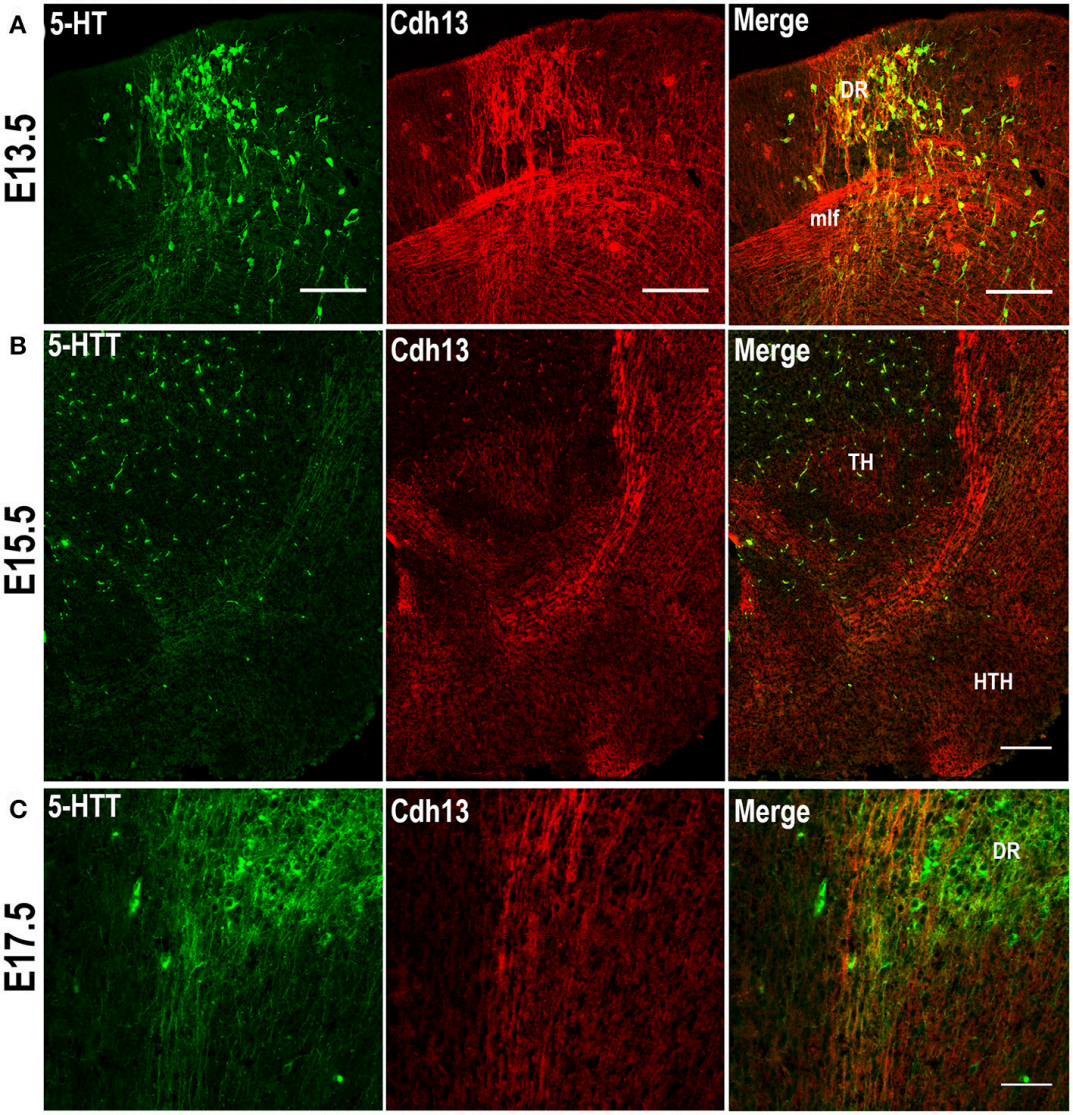
Cdh13 in 5-HT neurons of the dorsal raphe. **(A)** Cdh13 is highly expressed in the DR at E13.5. Cdh13 and 5-HTT-ir fibers follow the same trajectory from the hindbrain to the forebrain at E15.5 **(B)** and E17.5 **(C)**. Orientation: sagittal. Scale bars: 100 μm. DR, dorsal raphe; TH, thalamus; HTH, hypothalamus; mlf, medial longitudinal fasciculus.

The migratory pattern of CDH13 in embryonic stages closely follows the temporal progression of serotonergic fibers that originate from the rostral raphe nuclei (Figure 2A). These 5-HT neurons start extending their axons along the midbrain basal plate following the same path as Cdh13-positive fibers down the fasciculus retroflexus to the hypothalamus, the striatum, and the septum (Figures 2B,C). The 5-HT positive axons reach the forebrain at ~E16 (Bonnin et al., 2011), the time point at which the maximum of Cdh13 immunoreactivity is also pronounced in the cortex. Moreover, at E13.5 there is strong Cdh13 immunoreactivity in the region caudal to the MHB and above the medial longitudinal fasciculus (mlf), which corresponds with the developing DR.

### Cdh13 is expressed in 5-HT neurons in the dorsal raphe and in the developing prefrontal cortex

**Figure 3:**
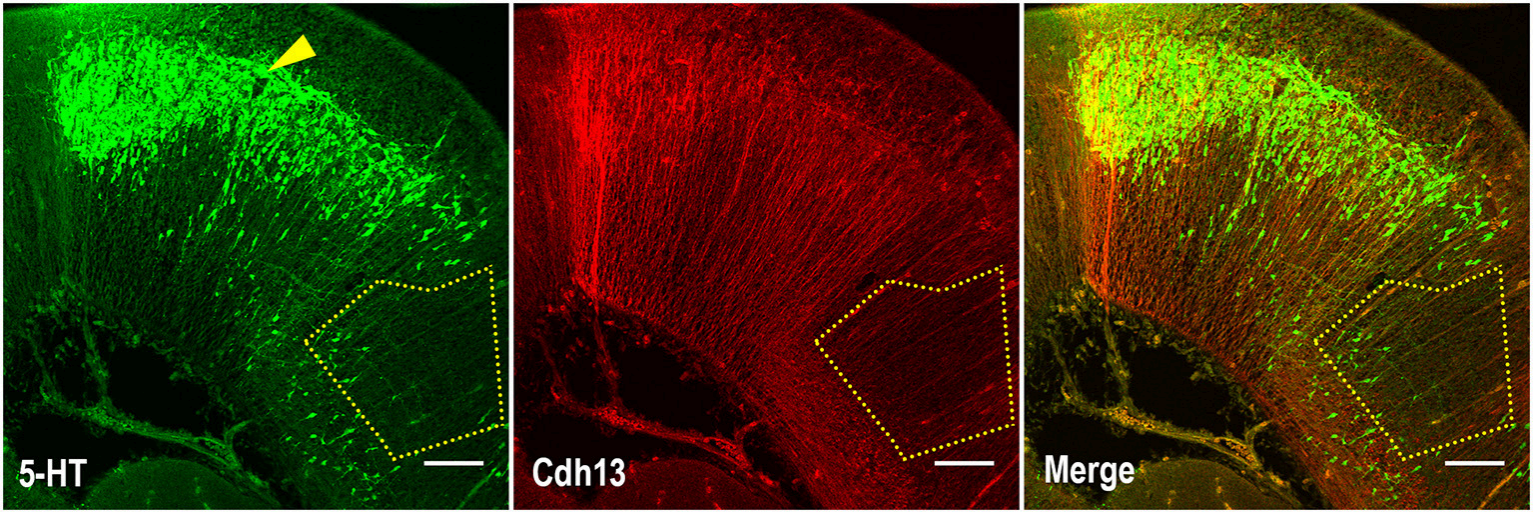
Cdh13 is not expressed in fibers in the median raphe. Representative images of the hindbrain region stained for Cdh13 and 5-HT. Cdh13 is not detected in the region corresponding to the developing MR (delimited area). Arrow indicates position of DR. Orientation: sagittal. Scale bar: 100 μm.

Based on the abundance of Cdh13 expression in the hindbrain at E13.5, the orientation and extension of serotonergic fibers co-localized with the Cdh13-expressing trajectory, and the detection of *Cdh13* mRNA in 5-HT neurons in the DR of adult mice (Rivero et al., 2013), we predicted that Cdh13 is associated with the development of the 5-HT system, more specifically, the structural configuration of the DR. Examination of the hindbrain region revealed that bundles of 5-HT neurons with migratory morphology located in the DR are accompanied by Cdh13-positive fibers (Figure 2A), whereas Cdh13 expression is largely absent in the developing MR (Figure 3).

**Figure 4:**
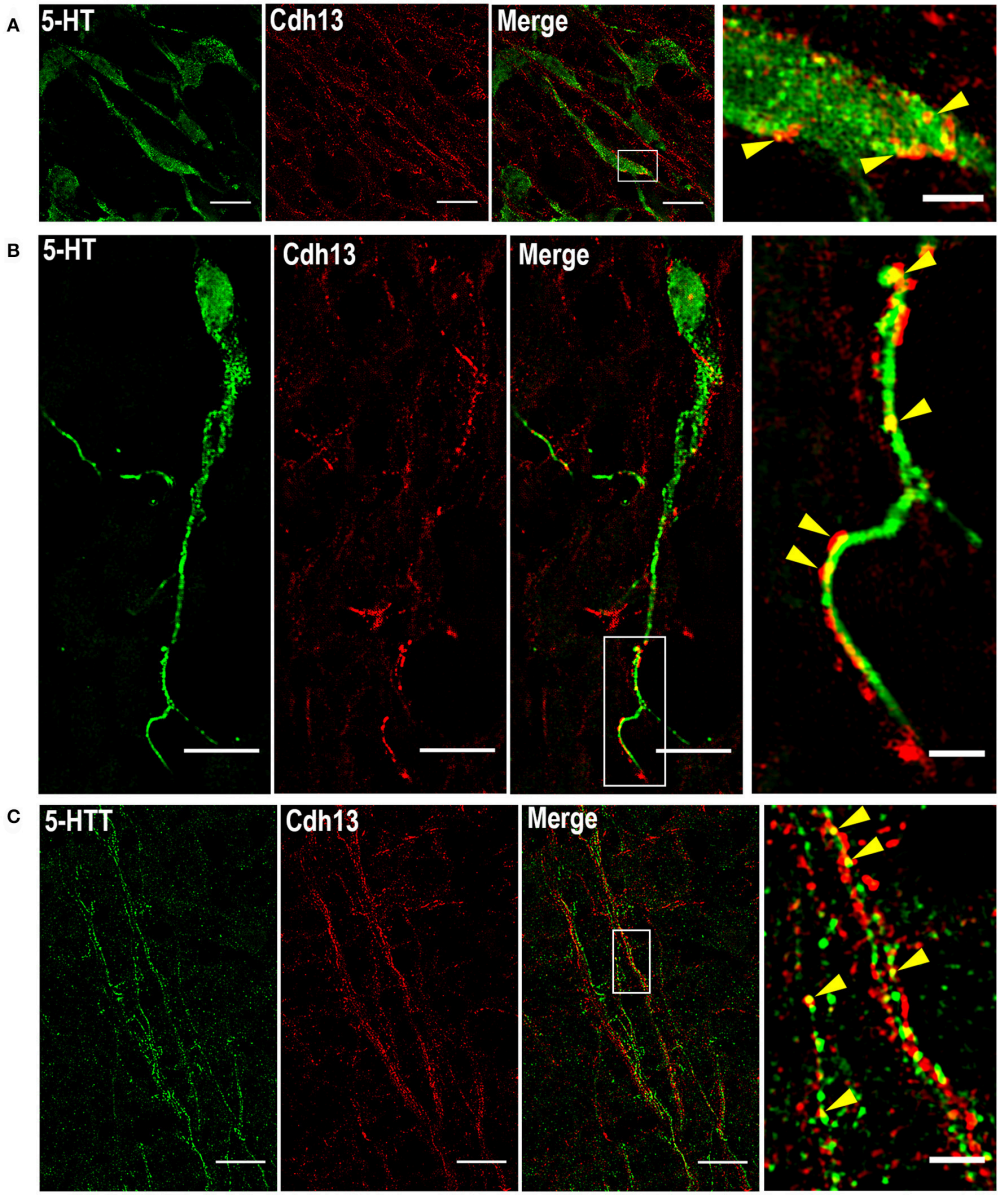
Subcellular localization of Cdh13 in DR 5-HT neurons. Using SIM, Cdh13 is detected in the soma **(A)** and neurites **(B)** of 5-HT positive neurons, and is also localized in 5-Htt positive fibers **(C)** (yellow arrows indicate points of colocalization). Orientation: sagittal. Scale bars in **(A)** 100 μm, in **(B,C)** 10 μm in full images, 2 μm in the magnified boxed regions.

In order to precisely determine the patterning of Cdh13 protein on these neurons, we applied the super-resolution technique SIM, which provides a two-fold increase in resolution compared to confocal microscopy (Gustafsson, 2000; Wegel et al., 2016). The use of SIM allowed us to identify Cdh13 in the soma of 5-HT positive neurons with its characteristic punctate pattern around the cell membrane and clustering at some discrete locations (Figure 4A). Moreover, Cdh13 is not only limited to the cell body, but is also present on the 5-HT neuron extension, with punctate immunoreactivity at some specific parts of the neurite (Figure 4B). Immunofluorescence staining for the 5-HT transporter (5-Htt/Sert) showed that in some sites Cdh13 co-localizes with 5-Htt, confirming that it is present on axons of these neurons (Figure 4C).

**Figure 5:**
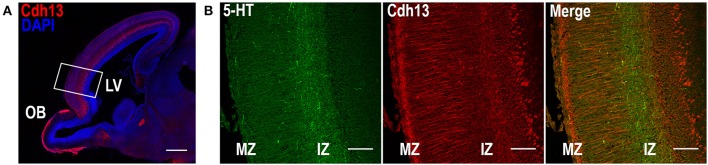
Cdh13 and 5-HT in prefrontal cortex at E17.5. **(A)** Cdh13 is expressed in cortex, including the PFC (delimited by a white rectangle). **(B)** In this region, 5-HT specific afferents innervate the intermediate (IZ) and marginal zones (MZ), both of which exhibit high immunoreactivity for Cdh13. Orientation: sagittal. Scale bar: 100 μm. IZ, intermediate zone; LV, lateral ventricle; MZ, marginal zone; OB, olfactory bulb.

Given the role of Cdh13 in neurite outgrowth and axonal guidance processes, we also examined Cdh13 in the cortex at E17.5. At this developmental stage, 5-HT afferents from the DR start to innervate the cortex at the IZ and the marginal zone (MZ), with both layers showing strong immunoreactivity for Cdh13 (Figure 5). This temporal coincidence suggests co-localization of Cdh13 with serotonergic fibers, prompting us to attempt further structural differentiation. Cdh13 is also present in the developing cortical plate, however the pattern of expression is different. In the IZ, Cdh13 is mostly just individual punctuates, probably because this layer is composed mostly of projections and terminals, while in layers which constitute the cortical plate the expression is more fiber-like.

### Cdh13 is present at intersecting points between 5-HT neurons and radial glial cells

**Figure 6:**
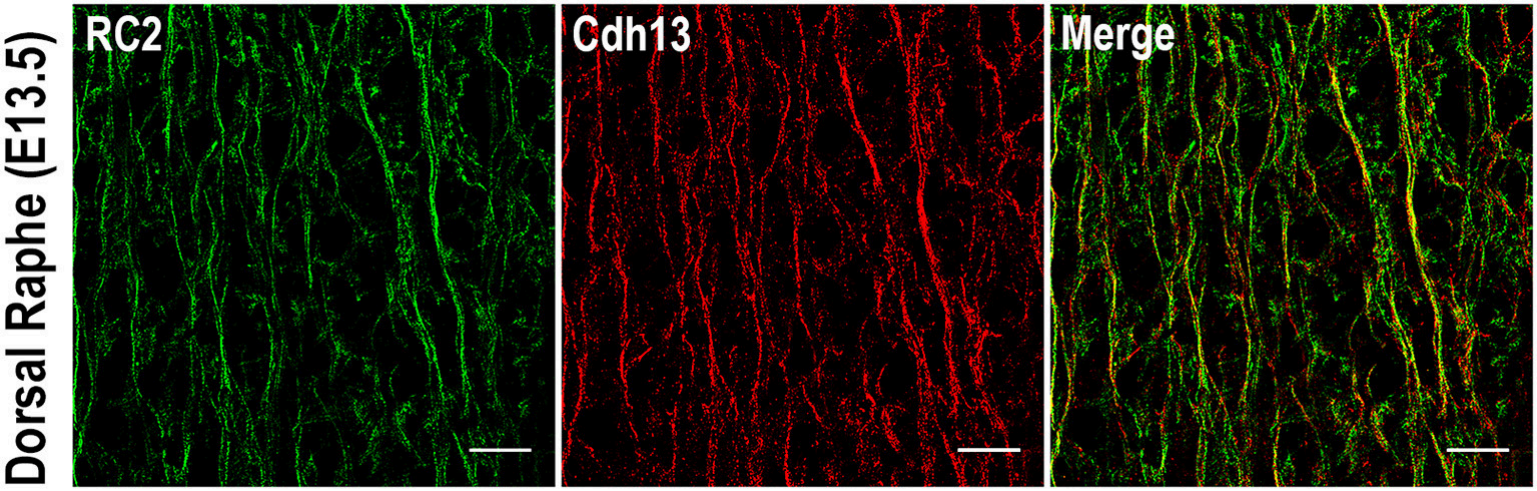
Cdh13 in radial glia cells in the dorsal raphe at E13.5. Cdh13 overlaps with RC2 immunoreactivity, a marker for radial glia cells. Orientation: sagittal. Scale bar: 100 μm.

The foregoing analysis of expression patterns revealed that Cdh13 is present not only on 5-HT positive neurons of the DR, but may be found also on neighboring cells. These Cdh13-immunoreactive cell extensions migrate in parallel and appear to create a scaffold for 5-HT neurons. The pattern suggests that Cdh13 is present on radial glial cells (RGCs). By using an isoform of the intermediate filament protein Nestin (RC2), a marker for RGCs (Park et al., 2009), we detected consistent co-localization between Cdh13 and RC2 immunoreactivity in the developing hindbrain (Figure 6). This overlap of expression confirms the identity of Cdh13-positive cell extensions as being distinct from serotonergic neurites. Taken together, these findings support the view that RGCs might be used by 5-HT neurons as a supportive framework during migration.

**Figure 7:**
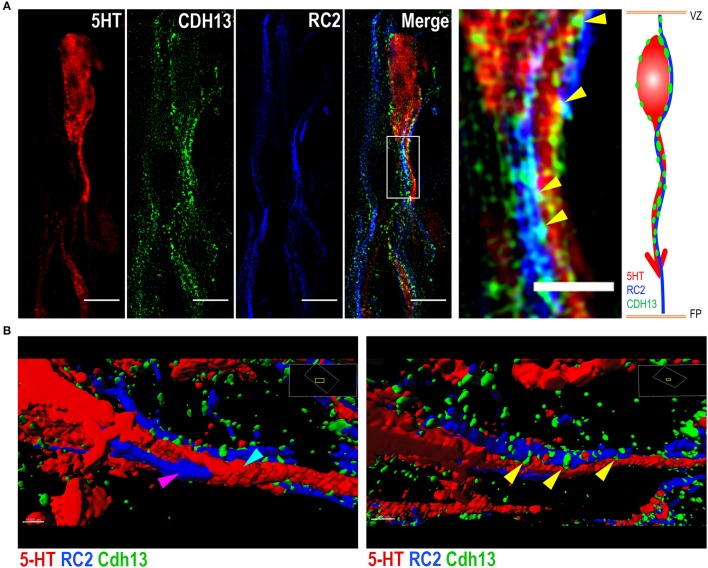
Cdh13 at points of intersection between 5-HT neurons and radial glial cells. Representative images of serotonergic fibers and radial glial cell (RGC) extensions triple-stained for 5-HT, RC2 and Cdh13. **(A,B)** Cdh13 (green) is present in both 5-HT neurons (red) and RGCs (blue), and at some points of intersection between these two cell types (yellow arrows). **(B)** Reconstruction of triple IF of 5-HT, RC2, and Cdh13 using Imaris. The 5-HT neuron (cyan arrow) is intertwined with the RGC fiber (magenta arrow). Cdh13 immunoreactivity is found at the interface between both cell types (Supplemental Videos 1 and 2). Orientation: sagittal. Scale bars in **(A)** 5 μm in full images, 2 μm in the magnified boxed region. FP, floor plate; VZ, ventricular zone.

The presence of Cdh13 on both 5-HT neurons and adjacent RGCs suggests a role for Cdh13 in RGC-mediated locomotion of 5-HT neurons. Triple immunofluorescence staining for 5-HT, RC2, and Cdh13 (Figure 7A) and reconstruction of spatial alignment of the three molecules (Figure 7B, Supplementary Movies 1, 2) yielded two findings in support of this mechanistic association: first, some 5-HT neurons in the DR are intertwined with RGCs, an organization suggesting that these neurons are using RGC structure as a physical guide to migrate. Second, Cdh13 is present at some points of intersection between the 5-HT specific and RGC types, both at the soma and the extending neurites, which indicates that cooperativity, possibly by homophilic interaction, may contribute to the migration process.

### Cdh13 deficiency affects the cell density of 5-HT neurons in the dorsal raphe and their innervation of the prefrontal cortex

**Figure 8:**
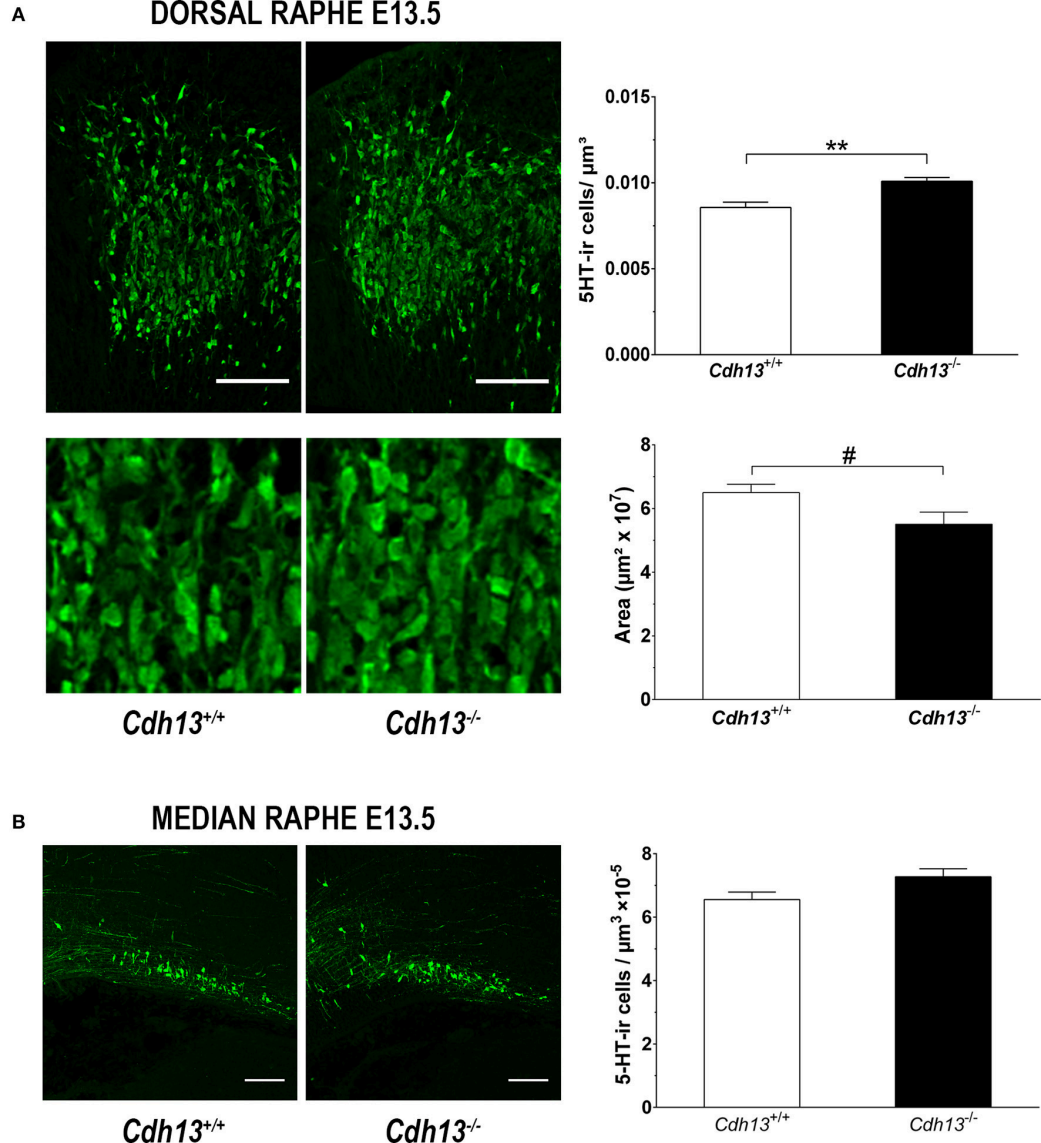
Cdh13 deficiency affects the cell density and area of the dorsal raphe, but not the median raphe, at E13.5. **(A)** Representative images of DR stained with 5-HT are shown. A significantly higher number of serotonergic neurons (*P* = 0.0014) within a predetermined area (397.5 μm^2^) and a trend toward reduced DR area (*P* = 0.051) is observed in Cdh13^−/−^ mice compared to wildtype controls. *n* = 8 per genotype. **(B)** Representative images of MR stained with 5-HT are shown. No significant differences in the density of serotonergic neurons in the MR between Cdh13^−/−^ and wildtype embryos is observed. *n* = 4 per genotype. Orientation: sagittal. Scale bar in **(A,B)**: 100 μm. Data are presented as mean ± s.e.m. ^#^*P* < 0.1, ^**^P < 0.01.

**Figure 9:**
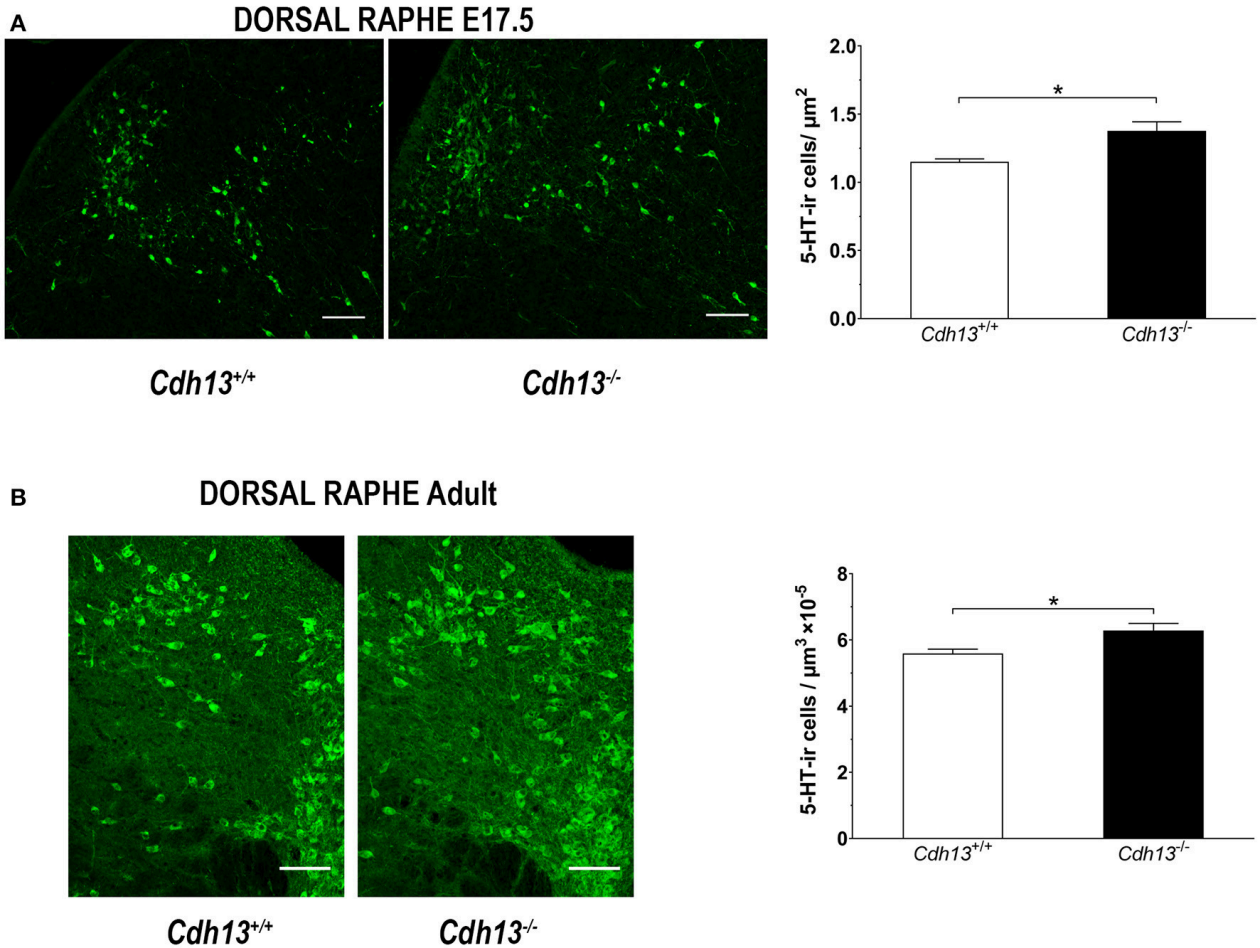
Cdh13 deficiency affects the cell density of the dorsal raphe at E17.5 and adulthood. Representative images of DR stained with 5-HT are shown for E17.5 **(A)** and adult **(B)** brains. A significantly higher density of serotonergic neurons is observed in *Cdh13*^−/−^ mice compared to wildtype controls at E17.5 **(A)**, *P* = 0.0227 and adult **(B)**, *P* = 0.0191 brains. *N* = *6* per genotype. Orientation in E17.5 sections: sagittal. Orientation in adult sections: coronal. Scale bar: 100 μm. Data are presented as mean ± s.e.m. ^*^P < 0.05.

**Figure 10:**
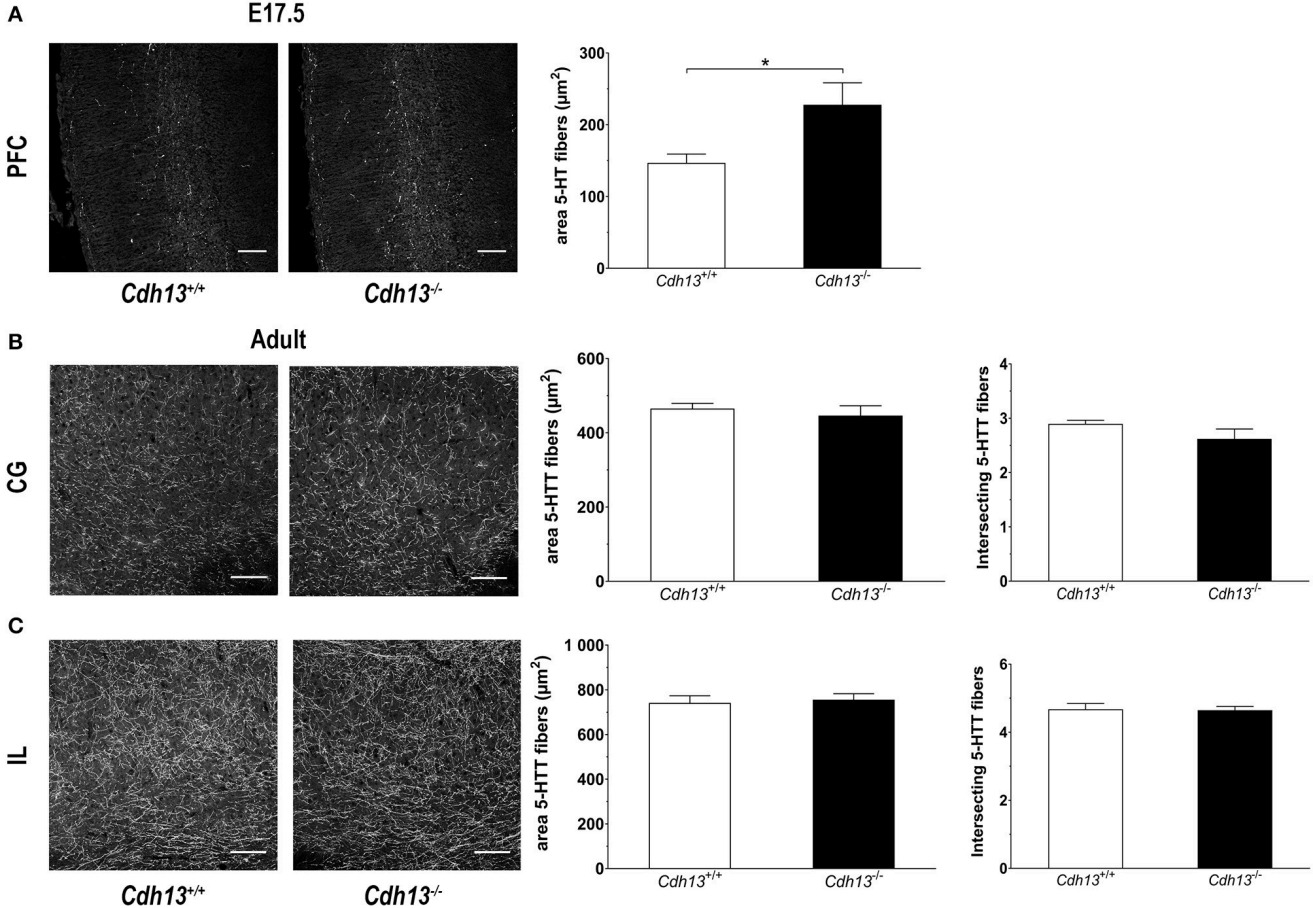
Cdh13 deficiency affects the serotonergic innervation of the prefrontal cortex at E17.5, but not in adulthood. **(A)** Representative images of PFC at E17.5 (stained with 5-HT) are shown. A larger area occupied by 5-HT-positive fibers is observed in *Cdh13*^−/−^ mice compared to wildtype controls (*P* = 0.042). *N* = 7 per genotype. **(B,C)** No significant differences in the serotonergic innervation (stained with 5-htt) of the IL and CG cortex, measured by two different approaches (see Methods section), is observed in adult brains. Orientation in E17.5 sections: sagittal. Orientation in adult sections: coronal. Scale bar in **(A,B)** 100 μm. Data are presented as mean ± s.e.m. ^*^P < 0.05.

The evidence for a role of Cdh13 in 5-HT neuron migration and fiber extension prompted us to study the effects of Cdh13 deficiency on the development of the DR-cortex 5-HT subsystem. We first investigated the effect of *Cdh13* inactivation in the formation of the DR by measuring the density of 5-HT positive neurons in *Cdh13*^−/−^ animals at E13.5, E17.5, and adult mice. At E13.5, we found an increase of DR 5-HT neuron density in *Cdh13*^−/−^ mice (*P* = 0.0014 vs. wildtype controls; Figure 8A), an effect that was not observed in the MR, where Cdh13 is not present (Figure 8B). Additionally, we observed a similar increase in the cell density of DR 5-HT positive neurons at E17.5 (*P* = 0.0227; Figure 9A) and in Tph2-ir neurons in the adult brain (*P* = 0.0191; Figure 9B), with a significant increase in *Cdh13*^−/−^ mice compared to *Cdh13*^+/+^ animals. Moreover, there is a tendency at E13.5 for the area occupied by DR 5-HT neurons in *Cdh13*^−/−^ mice to be smaller than in wildtype controls (*P* = 0.051; Figure 8A). However, this is not observed at E17.5 nor in the adult brain. We then investigated whether Cdh13 deficiency might also alter serotonergic innervation of the Prefrontal cortex (PFC) by determining the density of 5-HT positive fibers per defined area in the PFC at E17.5 and in the IL and CG cortices in the adult brain. We found an increase in serotonergic fiber density innervating at E17.5 in *Cdh13*^−/−^ embryos (*P* = 0.042 vs. wildtype controls; Figure 10A). However, the similar analysis in the adult brain, measuring the number as well as the area occupied by 5-HTT-ir fibers in the PFC did not yield any significant results (Figures 10B,C).

## Discussion

The development of the raphe nuclei and their projections to multiple areas is determined by both intrinsic and extrinsic molecular factors. In the present study, we identify a new molecular player in raphe neuron migration and development of forebrain projections. Our results demonstrate that Cdh13 delimits the MHB, a relevant organizer in the raphe nuclei formation, and that it is highly expressed on 5-HT specific neurons of the DR and on RGCs in this region. We additionally demonstrate that 5-HT neurons intertwine with these RGCs, a morphology that has not been observed in this cell type and suggest that these neurons undergo RGC-guided migration. Employing a super-resolution microscopy technique, we confirm the presence of Cdh13 at points of intersection between 5-HT neurons and RGCs. Furthermore, we show that Cdh13-deficient mice display increased cell densities in the DR at E13.5, E17.5, and in adulthood, as well as higher serotonergic innervation of the PFC at E17.5.

While previous studies have identified *Cdh13* mRNA in 5-HT neurons of the adult murine brain (Wylie et al., 2010; Rivero et al., 2013; Okaty et al., 2015), our findings confirm that Cdh13 protein is present in this cell type in earlier prenatal stages. In addition, our data indicate that during prenatal development Cdh13 expression is most strongly detected in the DR, one of the major sources of 5-HT innervation to the forebrain (Lesch and Waider, 2012). A structurally detailed expression pattern of Cdh13 on both the soma and extending neurites of 5-HT neurons was achieved by super-resolution microscopy. Cdh13 also co-localizes with the 5-Htt along 5-HT extensions suggesting that it may contribute to target recognition and synaptogenesis.

In addition, the observation that 5-HT neurons are intertwined with RGCs in the hindbrain led us to consider that RGCs might be implicated in their migratory and axon guidance processes. RGCs assist migration of specific neuronal cell types and the projection of their neurites at late prenatal stages of neurodevelopment (Gupta et al., 2002). While the RGCs-assisted migratory mechanism has almost exclusively been established for the telencephalon, with the exception of studies focused on radial migration of granule neurons in the cerebellum (Edmondson and Hatten, 1987; Adams et al., 2002), the interaction between RGCs and 5-HT neurons has not been previously described. Through a three-dimensional reconstruction, we observe the grasping and intertwining of 5-HT neurons to RGCs similar to the first reconstruction of migrating neurons through RGC-mediated locomotion (Rakic, 1978, 2003).

Further analysis of the unique expression pattern of Cdh13 in the hindbrain revealed a strong localization in RGCs. It was previously reported that Cdh2 (N-cadherin) is present on radial glia cell extensions and required for migration as well as axon formation of cortical neurons (Shikanai et al., 2011; Xu et al., 2015). Although both Cdh13 and Cdh2 were identified on RGCs, the distribution of their expression in the raphe nuclei appears to be distinct, with Cdh2 being more restricted to the MR region (Okaty et al., 2015). While the function of Cdh2 expressed in the hindbrain remains unknown and interaction between RGCs and 5-HT neurons was not previously described, our results indicate that Cdh13 is present at the interface between 5-HT neurons and RGCs and thus might take part in the developmental formation of the DR.

Hawthorne et al. (2010) reported that in early developmental stages, 5-HT neurons migrate across the neuroepithelium through dynamin-mediated somal translocation, without the aid of RGCs. However, it is likely that numerous additional regulators contribute to the fine-tuning of this complex process at later prenatal stages. Our results complement this finding by showing that at subsequent stages, 5-HT neurons are intertwined with RGCs, an organization that suggests that they undergo RGC-mediated locomotion. Therefore, the principle of migration observed for cortical neurons which states that somal translocation occurs at early phases of development when the neuroepithelium is relatively thin and that in later phases the migration is guided by RGCs (Gupta et al., 2002; Nadarajah and Parnavelas, 2002), might also hold true for 5-HT neurons in the hindbrain.

Regarding the contribution of Cdh13 to the formation of the DR, we observe that the absence of Cdh13 modifies the cell density of the DR. In Cdh13-deficient animals, there is a higher number of 5-HT-producing cells per μm^3^. This increase was not only observed at prenatal stages of neurodevelopment, but also in Cdh13^−/−^ adult mice. Previous findings reported that alterations in the MHB lead to a misplacement and/or reduction of 5-HT neurons (Brodski et al., 2003; Teraoka et al., 2004). Future studies in *Cdh13* knockout mice will have to address potential alterations in the MHB, as fibers rich in Cdh13 meet the caudal boundary of Otx2 in the hindbrain, marking a clear limit in the MHB. Therefore, changes in the MHB in Cdh13-deficient mice might explain the differences found in cell density of the DR.

Already 1 day after 5-HT neurons from the rostral cluster are born (~E10-E11) they start projecting toward forebrain region. The rostral raphe cluster, which comprises the DR and MR, projects mainly to the forebrain, including the PFC (Lidov and Molliver, 1982; Wallace and Lauder, 1983). These projections reach the developing cortex at around E16, traveling through the outermost layer known as the MZ and below the cortical plate in the IZ (Wallace and Lauder, 1983). Then, perpendicular axons begin to extend, innervating along the cortical plate (Vitalis et al., 2013). Alterations in molecules implicated in anterior-posterior orientation, midline guidance as well as axon elongation and maintenance, have been shown to affect projection of 5-HT axons (Kiyasova and Gaspar, 2011).

Previous studies implicating CDH13 in neurite outgrowth and axonal pathfinding (Ranscht and Bronner-Fraser, 1991; Fredette and Ranscht, 1994; Fredette et al., 1996; Bai et al., 2006) prompted us to analyze the effect of Cdh13 deficiency on the serotonergic innervation of the developing PFC. The PFC has been associated with higher-order brain functions, such as attentional processes, working memory, and social cognition (Miller and Cohen, 2001; Blakemore, 2008). Our results revealed that at E17.5, Cdh13 is strongly expressed throughout the developing cortex, including the PFC, a time point at which innervation of 5-HT afferents is also starting to develop (Vitalis et al., 2013). Our data indicate that in *Cdh13* knockout embryos, the innervation of serotonergic fibers in the PFC is increased. This is consistent with initial findings that Cdh13 functions as a negative regulator in neurite outgrowth (Ranscht and Bronner-Fraser, 1991; Fredette and Ranscht, 1994; Fredette et al., 1996; Bai et al., 2006). The mechanism underlying CDH13-mediated inhibition of axon growth is not well-understood. However, it is known that the growth associated protein-43 (GAP43), a phosphoprotein that is essential in the wiring of serotonergic circuits, interacts with neural cell adhesion molecules associated with neurite outgrowth and axon guidance, such as NCAM, L1 and CDH2 (Donovan et al., 2002). Likewise, CDH13 may also be among these cell adhesion molecules that GAP43 responds to, thus impacting projection of 5-HT neurons.

Disruption of *CDH13* by rare *de novo* and inherited deletions was linked to autism spectrum disorders (Sanders et al., 2011, 2015); Van der Burgt, personal communication). The impact of CDH13 dysfunction on formation of the 5-HT system specifically and brain function (Rivero et al., 2015) in general may be relevant for the etiopathogenesis of neurodevelopmental disorders. The relationship between altered 5-HT system function and these conditions, including autistic syndromes, has been amply discussed and reviewed (Gaspar et al., 2003; Lesch and Waider, 2012; Kiser et al., 2015; Lesch, 2016). Increased levels of whole blood 5-HT were identified in a subgroup of patients with autism (Anderson et al., 1987; Launay et al., 1988; Gabriele et al., 2014) as well as first-degree relatives of autistic patients (Leboyer et al., 1999). The elevation of peripheral 5-HT may be due to a disruption in the control of 5-HT production during development, since 5-HT synthesis capacity in the brain of children with autism increases between the ages of 2 and 15 to above the normal adult standard, while in healthy children initially elevated levels of cerebral 5-HT later decrease to adult values (Chugani et al., 1999). Our findings of altered 5-HT system development in Cdh13-deficient mice resulting in serotonergic hyperinnervation of the cortex is in line with the notion of a CDH13-driven pathogenetic mechanism affecting brain 5-HT system function in neurodevelopmental disorders.

An association of common *CDH13* variation with neurodevelopmental and psychiatric disorders, particularly ADHD (Lasky-Su et al., 2008; Lesch et al., 2008; Neale et al., 2008, 2010; Uhl et al., 2008a,b; Zhou et al., 2008; Treutlein et al., 2009; Lionel et al., 2011) and comorbid conditions, was reported. Common variation in genes coding for various components of 5-HT transmission (e.g., *HTR1B, SLC6A4/5-HTT, TPH2*) has previously been associated with a susceptibility to ADHD, which may epistatically interact with *CDH13* variants. Alterations in the 5-HT system, such as reduced brain 5-HT function and 5-HT hyperinnervation, have been identified in animal model for ADHD (Banerjee and Nandagopal, 2015). 5-HT is believed to contribute to ADHD and its treatment indirectly through its interaction with the dopamine system (Gainetdinov et al., 1999). A relationship between CDH13 and the brain dopamine system has been recently described (Drgonova et al., 2016). *Cdh13*^−/−^ mice displayed alterations in the cortex including increased dopaminergic innervation, reduced levels of dopamine and an altered dopamine/metabolites ratio. In addition to alterations in 5-HT system function, dysregulation of dopaminergic signaling has consistently been implicated in ADHD. This combined involvement of at least two monoaminergic systems may thus represent a basis for the pervasive pathogenetic mechanisms of ADHD.

In conclusion, our study provides evidence for a role of CDH13 in the development of the serotonergic system in early embryonic stages. Moreover, dysregulation of Cdh13 expression during development may contribute to alterations in 5-HT neuron migration and density in the DR as well as impaired organization of serotonergic innervation and circuit formation in frontal cortex, thus impacting cognitive function, which is frequently impaired in neurodevelopmental disorders.

## Author contributions

AF, OR, MS, and KL conceived and designed research; AF, OR, SW, DK, YG, HK, and LP performed research; AF, SW, YG, and HK analyzed data; AF, OR, SW, JW, PG, CJ, FE, TR, RB, MS, and KL interpreted data; PG, CJ, TR, and RB assisted with data analysis and interpretation; AF, OR, SW, DK, JW, TR, RB, MS, and KL wrote the manuscript.

### Conflict of interest statement

The authors declare that the research was conducted in the absence of any commercial or financial relationships that could be construed as a potential conflict of interest.

## Supplementary material

Supplementary Figure 1 Counting of 5-HTT-ir fibers in the prefrontal cortex. **(A)** Illustrations of selected areas used for the counting of 5-HTT-ir fibers in the infralimbic (IL) and cingulate (CG) cortices. A grid of 70^*^70 μm (red) containing three yellow lines with intervals of 17.5 μm was placed over the binary image. **(B)** Example of the intensity profile of the measurements from one square, each peak represents a detected fiber.

Supplementary Movies 1 and 2 3D reconstruction of triple IF of 5-HT, RC2, and Cdh13. Movies illustrating 3D reconstruction using Imaris. 5-HT-positive neurons are labeled in blue, RC2-fibers are labeled in red and Cdh13 immunofluorescence is represented in green.

## Abbreviations

ADHD: Attention-deficit/hyperactivity disorder
CG: Cingulate cortex
CNV: Copy number variants
DR: Dorsal raphe
E: Embryonic day
IL: Infralimbic cortex
IZ: Intermediate zone
MHB: Mid-hindbrain boundary
MR: Median raphe
MZ: Marginal zone
PFC: Prefrontal cortex
RGCs: Radial glial cells
SIM: Structured illumination microscopy.

## References

[B1] Adams N. C. Tomoda T. Cooper M. Dietz G. Hatten M. E. (2002). Mice that lack astrotactin have slowed neuronal migration. Development 129, 965–972. 11861479 10.1242/dev.129.4.965

[B2] Alonso A. Merchan P. Sandoval J. E. Sanchez-Arrones L. Garcia-Cazorla A. Artuch R.. (2013). Development of the serotonergic cells in murine raphe nuclei and their relations with rhombomeric domains. Brain Struct. Funct. 218, 1229–1277. 10.1007/s00429-012-0456-8 23052546 PMC3748323

[B3] Anderson G. M. Freedman D. X. Cohen D. J. Volkmar F. R. Hoder E. L. McPhedran P.. (1987). Whole blood serotonin in autistic and normal subjects. J. Child Psychol. Psychiatry 28, 885–900. 10.1111/j.1469-7610.1987.tb00677.x 3436995

[B4] Arias-Vasquez A. Altink M. E. Rommelse N. N. Slaats-Willemse D. I. Buschgens C. J. Fliers E. A.. (2011). CDH13 is associated with working memory performance in attention deficit/hyperactivity disorder. Genes Brain Behav. 10, 844–851. 10.1111/j.1601-183X.2011.00724.x 21815997

[B5] Bai S. Ghoshal K. Jacob S. T. (2006). Identification of T-cadherin as a novel target of DNA methyltransferase 3B and its role in the suppression of nerve growth factor-mediated neurite outgrowth in PC12 cells. J. Biol. Chem. 281, 13604–13611. 10.1074/jbc.M513278200 16537533 PMC2241734

[B6] Banerjee E. Nandagopal K. (2015). Does serotonin deficit mediate susceptibility to ADHD? Neurochem. Int. 82, 52–68. 10.1016/j.neuint.2015.02.001 25684070

[B7] Blakemore S. J. (2008). The social brain in adolescence. Nat. Rev. Neurosci. 9, 267–277. 10.1038/nrn2353 18354399

[B8] Bonnin A. Levitt P. (2011). Fetal, maternal, and placental sources of serotonin and new implications for developmental programming of the brain. Neuroscience 197, 1–7. 10.1016/j.neuroscience.2011.10.005 22001683 PMC3225275

[B9] Bonnin A. Goeden N. Chen K. Wilson M. L. King J. Shih J. C.. (2011). A transient placental source of serotonin for the fetal forebrain. Nature 472, 347–350. 10.1038/nature09972 21512572 PMC3084180

[B10] Borglum A. D. Demontis D. Grove J. Pallesen J. Hollegaard M. V. Pedersen C. B.. (2014). Genome-wide study of association and interaction with maternal cytomegalovirus infection suggests new schizophrenia loci. Mol. Psychiatry 19, 325–333. 10.1038/mp.2013.2 23358160 PMC3932405

[B11] Boylan C. B. Blue M. E. Hohmann C. F. (2007). Modeling early cortical serotonergic deficits in autism. Behav. Brain Res. 176, 94–108. 10.1016/j.bbr.2006.08.026 17034875 PMC2570481

[B12] Brodski C. Weisenhorn D. M. Signore M. Sillaber I. Oesterheld M. Broccoli V.. (2003). Location and size of dopaminergic and serotonergic cell populations are controlled by the position of the midbrain-hindbrain organizer. J. Neurosci. 23, 4199–4207. 12764108 10.1523/JNEUROSCI.23-10-04199.2003 PMC6741088

[B13] Chugani D. C. Muzik O. Behen M. Rothermel R. Janisse J. J. Lee J.. (1999). Developmental changes in brain serotonin synthesis capacity in autistic and nonautistic children. Ann. Neurol. 45, 287–295. 10.1002/1531-8249(199903)45:3<287::AID-ANA3>3.0.CO;2-9 10072042

[B14] Ciatto C. Bahna F. Zampieri N. VanSteenhouse H. C. Katsamba P. S. Ahlsen G.. (2010). T-cadherin structures reveal a novel adhesive binding mechanism. Nat. Struct. Mol. Biol. 17, 339–347. 10.1038/nsmb.1781 20190755 PMC2873897

[B15] Daubert E. A. Condron B. G. (2010). Serotonin: a regulator of neuronal morphology and circuitry. Trends Neurosci. 33, 424–434. 10.1016/j.tins.2010.05.005 20561690 PMC2929308

[B16] Donovan S. L. Mamounas L. A. Andrews A. M. Blue M. E. McCasland J. S. (2002). GAP-43 is critical for normal development of the serotonergic innervation in forebrain. J. Neurosci. 22, 3543–3552. 10.1080/08990220701830696 11978831 PMC6758352

[B17] Drgonova J. Walther D. Hartstein G. L. Bukhari M. O. Baumann M. H. Katz J.. (2016). Cadherin 13: human cis-regulation and selectively-altered addiction phenotypes and cerebral cortical dopamine in knockout mice. Mol. Med. 22, 537–547. 10.2119/molmed.2015.00170 27579475 PMC5082297

[B18] Edmondson J. C. Hatten M. E. (1987). Glial-guided granule neuron migration *in vitro*: a high-resolution time-lapse video microscopic study. J. Neurosci. 7, 1928–1934. 3598656 10.1523/JNEUROSCI.07-06-01928.1987 PMC6568879

[B19] Fredette B. J. Ranscht B. (1994). T-cadherin expression delineates specific regions of the developing motor axon-hindlimb projection pathway. J. Neurosci. 14, 7331–7346. 7996179 10.1523/JNEUROSCI.14-12-07331.1994 PMC6576899

[B20] Fredette B. J. Miller J. Ranscht B. (1996). Inhibition of motor axon growth by T-cadherin substrata. Development 122, 3163–3171. 8898229 10.1242/dev.122.10.3163

[B21] Gabriele S. Sacco R. Persico A. M. (2014). Blood serotonin levels in autism spectrum disorder: a systematic review and meta-analysis. Eur. Neuropsychopharmacol. 24, 919–929. 10.1016/j.euroneuro.2014.02.004 24613076

[B22] Gainetdinov R. R. Wetsel W. C. Jones S. R. Levin E. D. Jaber M. Caron M. G. (1999). Role of serotonin in the paradoxical calming effect of psychostimulants on hyperactivity. Science 283, 397–401. 10.1126/science.283.5400.397 9888856

[B23] Gaspar P. Cases O. Maroteaux L. (2003). The developmental role of serotonin: news from mouse molecular genetics. Nat. Rev. Neurosci. 4, 1002–1012. 10.1038/nrn1256 14618156

[B24] Gomez C. Brinon J. G. Orio L. Colado M. I. Lawrence A. J. Zhou F. C.. (2007). Changes in the serotonergic system in the main olfactory bulb of rats unilaterally deprived from birth to adulthood. J. Neurochem. 100, 924–938. 10.1111/j.1471-4159.2006.04229.x 17266734

[B25] Gupta A. Tsai L. H. Wynshaw-Boris A. (2002). Life is a journey: a genetic look at neocortical development. Nat. Rev. Genet. 3, 342–355. 10.1038/nrg799 11988760

[B26] Gustafsson M. G. (2000). Surpassing the lateral resolution limit by a factor of two using structured illumination microscopy. J. Microsc. 198(Pt 2), 82–87. 10.1046/j.1365-2818.2000.00710.x 10810003

[B27] Gutknecht L. Kriegebaum C. Waider J. Schmitt A. Lesch K. P. (2009). Spatio-temporal expression of tryptophan hydroxylase isoforms in murine and human brain: convergent data from Tph2 knockout mice. Eur. Neuropsychopharmacol. 19, 266–282. 10.1016/j.euroneuro.2008.12.005 19181488

[B28] Gutknecht L. Waider J. Kraft S. Kriegebaum C. Holtmann B. Reif A.. (2008). Deficiency of brain 5-HT synthesis but serotonergic neuron formation in Tph2 knockout mice. J Neural Transm. 115, 1127–1132. 10.1007/s00702-008-0096-6 18665319

[B29] Halbleib J. M. Nelson W. J. (2006). Cadherins in development: cell adhesion, sorting, and tissue morphogenesis. Genes Dev. 20, 3199–3214. 10.1101/gad.1486806 17158740

[B30] Hawthorne A. L. Wylie C. J. Landmesser L. T. Deneris E. S. Silver J. (2010). Serotonergic neurons migrate radially through the neuroepithelium by dynamin-mediated somal translocation. J. Neurosci. 30, 420–430. 10.1523/JNEUROSCI.2333-09.2010 20071506 PMC2855244

[B31] Hayano Y. Zhao H. Kobayashi H. Takeuchi K. Norioka S. Yamamoto N. (2014). The role of T-cadherin in axonal pathway formation in neocortical circuits. Development 141, 4784–4793. 10.1242/dev.108290 25468941

[B32] Kiser D. P. Rivero O. Lesch K. P. (2015). Annual research review: The (epi)genetics of neurodevelopmental disorders in the era of whole-genome sequencing–unveiling the dark matter. J. Child Psychol. Psychiatry 56, 278–295. 10.1111/jcpp.12392 25677560

[B33] Kiyasova V. Gaspar P. (2011). Development of raphe serotonin neurons from specification to guidance. Eur. J. Neurosci. 34, 1553–1562. 10.1111/j.1460-9568.2011.07910.x 22103413

[B34] Lasky-Su J. Neale B. M. Franke B. Anney R. J. Zhou K. Maller J. B.. (2008). Genome-wide association scan of quantitative traits for attention deficit hyperactivity disorder identifies novel associations and confirms candidate gene associations. Am. J. Med. Genet. B Neuropsychiatr. Genet. 147B, 1345–1354. 10.1002/ajmg.b.30867 18821565

[B35] Launay J. M. Ferrari P. Haimart M. Bursztejn C. Tabuteau F. Braconnier A.. (1988). Serotonin metabolism and other biochemical parameters in infantile autism. A controlled study of 22 autistic children. Neuropsychobiology 20, 1–11. 10.1159/000118465 2466221

[B36] Leboyer M. Philippe A. Bouvard M. Guilloud-Bataille M. Bondoux D. Tabuteau F.. (1999). Whole blood serotonin and plasma beta-endorphin in autistic probands and their first-degree relatives. Biol. Psychiatry 45, 158–163. 10.1016/S0006-3223(97)00532-5 9951562

[B37] Lendahl U. Zimmerman L. B. McKay R. D. (1990). CNS stem cells express a new class of intermediate filament protein. Cell 60, 585–595. 1689217 10.1016/0092-8674(90)90662-x

[B38] Lesch K. P. (2016). Maturing insights into the genetic architecture of neurodevelopmental disorders - from common and rare variant interplay to precision psychiatry. J. Child Psychol. Psychiatry 57, 659–661. 10.1111/jcpp.12574 27192951

[B39] Lesch K. P. Waider J. (2012). Serotonin in the modulation of neural plasticity and networks: implications for neurodevelopmental disorders. Neuron 76, 175–191. 10.1016/j.neuron.2012.09.013 23040814

[B40] Lesch K. P. Timmesfeld N. Renner T. J. Halperin R. Roser C. Nguyen T. T.. (2008). Molecular genetics of adult ADHD: converging evidence from genome-wide association and extended pedigree linkage studies. J. Neural Transm. 115, 1573–1585. 10.1007/s00702-008-0119-3 18839057

[B41] Lidov H. G. Molliver M. E. (1982). An immunohistochemical study of serotonin neuron development in the rat: ascending pathways and terminal fields. Brain Res. Bull. 8, 389–430. 10.1016/0361-9230(82)90077-6 6178481

[B42] Li J. Y. Joyner A. L. (2001). Otx2 and Gbx2 are required for refinement and not induction of mid-hindbrain gene expression. Development 128, 4979–4991. 11748135 10.1242/dev.128.24.4979

[B43] Lionel A. C. Crosbie J. Barbosa N. Goodale T. Thiruvahindrapuram B. Rickaby J.. (2011). Rare copy number variation discovery and cross-disorder comparisons identify risk genes for ADHD. Sci. Transl. Med. 3:95ra75. 10.1126/scitranslmed.3002464 21832240

[B44] McNamara I. M. Borella A. W. Bialowas L. A. Whitaker-Azmitia P. M. (2008). Further studies in the developmental hyperserotonemia model (DHS) of autism: social, behavioral and peptide changes. Brain Res. 1189, 203–214. 10.1016/j.brainres.2007.10.063 18062943

[B45] Miller E. K. Cohen J. D. (2001). An integrative theory of prefrontal cortex function. Annu. Rev. Neurosci. 24, 167–202. 10.1146/annurev.neuro.24.1.167 11283309

[B46] Nadarajah B. Parnavelas J. G. (2002). Modes of neuronal migration in the developing cerebral cortex. Nat. Rev. Neurosci. 3, 423–432. 10.1038/nrn845 12042877

[B47] Neale B. M. Lasky-Su J. Anney R. Franke B. Zhou K. Maller J. B. (2008). Genome-wide association scan of attention deficit hyperactivity disorder. Am. J. Med. Genet. B Neuropsychiatr. Genet. 147B, 1337–1344. 10.1002/ajmg.b.30866 18980221 PMC2831205

[B48] Neale B. M. Medland S. Ripke S. Anney R. J. Asherson P. Buitelaar J.. (2010). Case-control genome-wide association study of attention-deficit/hyperactivity disorder. J. Am. Acad. Child Adolesc. Psychiatry 49, 906–920. 10.1016/j.jaac.2010.06.007 20732627 PMC2928577

[B49] Okaty B. W. Freret M. E. Rood B. D. Brust R. D. Hennessy M. L. deBairos D.. (2015). Multi-scale molecular deconstruction of the serotonin neuron system. Neuron 88, 774–791. 10.1016/j.neuron.2015.10.007 26549332 PMC4809055

[B50] Paradis S. Harrar D. B. Lin Y. Koon A. C. Hauser J. L. Griffith E. C.. (2007). An RNAi-based approach identifies molecules required for glutamatergic and GABAergic synapse development. Neuron 53, 217–232. 10.1016/j.neuron.2006.12.012 17224404 PMC1950560

[B51] Park D. Xiang A. P. Zhang L. Mao F. F. Walton N. M. Choi S. S.. (2009). The radial glia antibody RC2 recognizes a protein encoded by Nestin. Biochem. Biophys. Res. Commun. 382, 588–592. 10.1016/j.bbrc.2009.03.074 19302980

[B52] Rakic P. (1978). Neuronal migration and contact guidance in the primate telencephalon. Postgrad. Med. J. 54(Suppl. 1), 25–40. 364453

[B53] Rakic P. (2003). Elusive radial glial cells: historical and evolutionary perspective. Glia 43, 19–32. 10.1002/glia.10244 12761862

[B54] Ranscht B. Bronner-Fraser M. (1991). T-cadherin expression alternates with migrating neural crest cells in the trunk of the avian embryo. Development 111, 15–22. 1707785 10.1242/dev.111.1.15

[B55] Ranscht B. Dours-Zimmermann M. T. (1991). T-cadherin, a novel cadherin cell adhesion molecule in the nervous system lacks the conserved cytoplasmic region. Neuron 7, 391–402. 10.1016/0896-6273(91)90291-7 1654948

[B56] Redies C. (1995). Cadherin expression in the developing vertebrate CNS: from neuromeres to brain nuclei and neural circuits. Exp. Cell Res. 220, 243–256. 10.1006/excr.1995.1313 7556431

[B57] Rivero O. Selten M. M. Sich S. Popp S. Bacmeister L. Amendola E.. (2015). Cadherin-13, a risk gene for ADHD and comorbid disorders, impacts GABAergic function in hippocampus and cognition. Transl. Psychiatry 5:Pe655. 10.1038/tp.2015.152 26460479 PMC4930129

[B58] Rivero O. Sich S. Popp S. Schmitt A. Franke B. Lesch K. P. (2013). Impact of the ADHD-susceptibility gene CDH13 on development and function of brain networks. Eur. Neuropsychopharmacol. 23, 492–507. 10.1016/j.euroneuro.2012.06.009 22795700

[B59] Sanders S. J. Ercan-Sencicek A. G. Hus V. Luo R. Murtha M. T. Moreno-De-Luca D.. (2011). Multiple recurrent *de novo* CNVs, including duplications of the 7q11.23 Williams syndrome region, are strongly associated with autism. Neuron 70, 863–885. 10.1016/j.neuron.2011.05.002 21658581 PMC3939065

[B60] Sanders S. J. He X. Willsey A. J. Ercan-Sencicek A. G. Samocha K. E. Cicek A. E.. (2015). Insights into autism spectrum disorder genomic architecture and biology from 71 risk loci. Neuron 87, 1215–1233. 10.1016/j.neuron.2015.09.016 26402605 PMC4624267

[B61] Schneider C. A. Rasband W. S. Eliceiri K. W. (2012). NIH Image to ImageJ: 25 years of image analysis. Nat. Methods 9, 671–675. 10.1038/nmeth.2089 22930834 PMC5554542

[B62] Shikanai M. Nakajima K. Kawauchi T. (2011). N-cadherin regulates radial glial fiber-dependent migration of cortical locomoting neurons. Commun. Integr. Biol. 4, 326–330. 10.4161/cib.4.3.14886 21980571 PMC3187899

[B63] Sibille E. Wang Y. Joeyen-Waldorf J. Gaiteri C. Surget A. Oh S.. (2009). A molecular signature of depression in the amygdala. Am. J. Psychiatry 166, 1011–1024. 10.1176/appi.ajp.2009.08121760 19605536 PMC2882057

[B64] Takeichi M. (2007). The cadherin superfamily in neuronal connections and interactions. Nat. Rev. Neurosci. 8, 11–20. 10.1038/nrn2043 17133224

[B65] Takeuchi T. Misaki A. Liang S. B. Tachibana A. Hayashi N. Sonobe H.. (2000). Expression of T-cadherin (CDH13, H-Cadherin) in human brain and its characteristics as a negative growth regulator of epidermal growth factor in neuroblastoma cells. J. Neurochem. 74, 1489–1497. 10.1046/j.1471-4159.2000.0741489.x 10737605

[B66] Teissier A. Soiza-Reilly M. Gaspar P. (2017). Refining the role of 5-HT in postnatal development of brain circuits. Front. Cell. Neurosci. 11:139. 10.3389/fncel.2017.00139 28588453 PMC5440475

[B67] Teraoka H. Russell C. Regan J. Chandrasekhar A. Concha M. L. Yokoyama R.. (2004). Hedgehog and Fgf signaling pathways regulate the development of tphR-expressing serotonergic raphe neurons in zebrafish embryos. J. Neurobiol. 60, 275–288. 10.1002/neu.20023 15281067 PMC2789256

[B68] Terracciano A. Tanaka T. Sutin A. R. Sanna S. Deiana B. Lai S.. (2010). Genome-wide association scan of trait depression. Biol. Psychiatry 68, 811–817. 10.1016/j.biopsych.2010.06.030 20800221 PMC2955852

[B69] Tiihonen J. Rautiainen M. R. Ollila H. M. Repo-Tiihonen E. Virkkunen M. Palotie A.. (2015). Genetic background of extreme violent behavior. Mol. Psychiatry 20, 786–792. 10.1038/mp.2014.130 25349169 PMC4776744

[B70] Treutlein J. Cichon S. Ridinger M. Wodarz N. Soyka M. Zill P.. (2009). Genome-wide association study of alcohol dependence. Arch. Gen. Psychiatry 66, 773–784. 10.1001/archgenpsychiatry.2009.83 19581569 PMC4229246

[B71] Uhl G. R. Drgon T. Johnson C. Li C. Y. Contoreggi C. Hess J.. (2008a). Molecular genetics of addiction and related heritable phenotypes: genome-wide association approaches identify connectivity constellation and drug target genes with pleiotropic effects. Ann. N. Y. Acad. Sci. 1141, 318–381. 10.1196/annals.1441.018 18991966 PMC3922196

[B72] Uhl G. R. Drgon T. Liu Q. R. Johnson C. Walther D. Komiyama T.. (2008b). Genome-wide association for methamphetamine dependence: convergent results from 2 samples. Arch. Gen. Psychiatry 65, 345–355. 10.1001/archpsyc.65.3.345 18316681

[B73] Vitalis T. Ansorge M. S. Dayer A. G. (2013). Serotonin homeostasis and serotonin receptors as actors of cortical construction: special attention to the 5-HT3A and 5-HT6 receptor subtypes. Front. Cell. Neurosci. 7:93. 10.3389/fncel.2013.00093 23801939 PMC3686152

[B74] Vitalis T. Cases O. Passemard S. Callebert J. Parnavelas J. G. (2007). Embryonic depletion of serotonin affects cortical development. Eur. J. Neurosci. 26, 331–344. 10.1111/j.1460-9568.2007.05661.x 17650110

[B75] Wallace J. A. Lauder J. M. (1983). Development of the serotonergic system in the rat embryo: an immunocytochemical study. Brain Res. Bull. 10, 459–479. 10.1016/0361-9230(83)90144-2 6344960

[B76] Wegel E. Gohler A. Lagerholm B. C. Wainman A. Uphoff S. Kaufmann R.. (2016). Imaging cellular structures in super-resolution with SIM, STED and localisation microscopy: a practical comparison. Sci. Rep. 6:27290. 10.1038/srep27290 27264341 PMC4893670

[B77] Whittle N. Sartori S. B. Dierssen M. Lubec G. Singewald N. (2007). Fetal Down syndrome brains exhibit aberrant levels of neurotransmitters critical for normal brain development. Pediatrics 120, e1465–1471. 10.1542/peds.2006-3448 17998315

[B78] Wylie C. J. Hendricks T. J. Zhang B. Wang L. Lu P. Leahy P.. (2010). Distinct transcriptomes define rostral and caudal serotonin neurons. J. Neurosci. 30, 670–684. 10.1523/JNEUROSCI.4656-09.2010 20071532 PMC3403750

[B79] Xu C. Funahashi Y. Watanabe T. Takano T. Nakamuta S. Namba T.. (2015). Radial glial cell-neuron interaction directs axon formation at the opposite side of the neuron from the contact site. J. Neurosci. 35, 14517–14532. 10.1523/JNEUROSCI.1266-15.2015 26511243 PMC4623227

[B80] Xu W. Cohen-Woods S. Chen Q. Noor A. Knight J. Hosang G.. (2014). Genome-wide association study of bipolar disorder in Canadian and UK populations corroborates disease loci including SYNE1 and CSMD1. BMC Med. Genet. 15:2. 10.1186/1471-2350-15-2 24387768 PMC3901032

[B81] Ye W. Shimamura K. Rubenstein J. L. Hynes M. A. Rosenthal A. (1998). FGF and Shh signals control dopaminergic and serotonergic cell fate in the anterior neural plate. Cell 93, 755–766. 10.1016/S0092-8674(00)81437-3 9630220

[B82] Zhou K. Dempfle A. Arcos-Burgos M. Bakker S. C. Banaschewski T. Biederman J.. (2008). Meta-analysis of genome-wide linkage scans of attention deficit hyperactivity disorder. Am. J. Med. Genet. B Neuropsychiatr. Genet. 147B, 1392–1398. 10.1002/ajmg.b.30878 18988193 PMC2890047

