# Supplementary figures and images for: Cadherin-13 Deficiency Increases Dorsal Raphe 5-HT Neuron Density and Prefrontal Cortex Innervation in the Mouse Brain
Source: Front Cell Neurosci. 2017 Sep 26;11:307. doi: 10.3389/fncel.2017.00307 (PMC5623013; doi:10.3389/fncel.2017.00307)

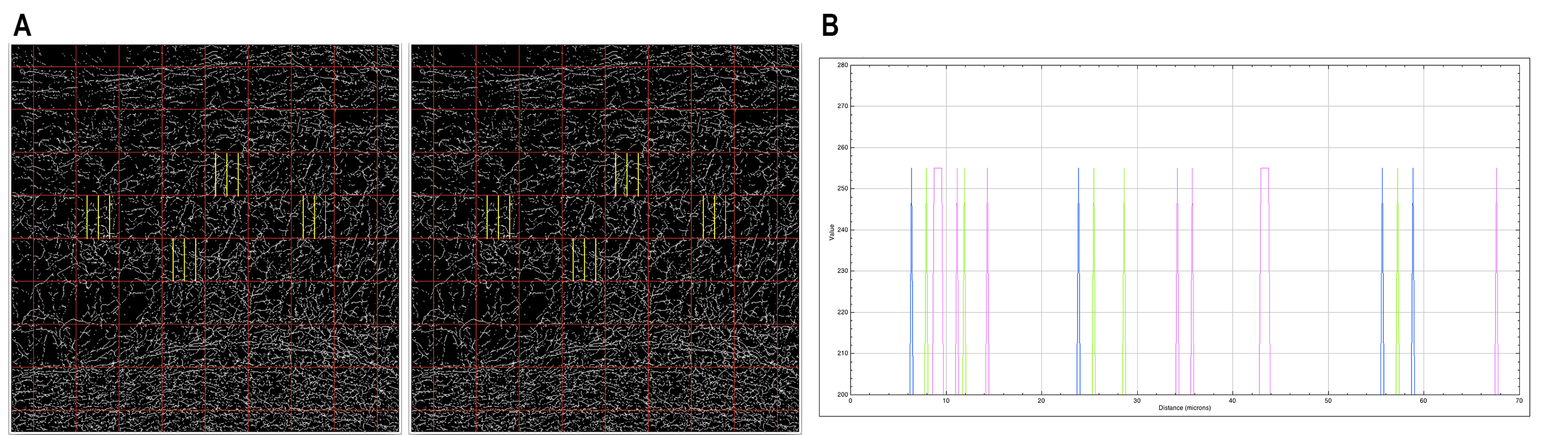

Supplement: Supplementary Figure 1 — Counting of 5-HTT-ir fibers in the prefrontal cortex. (A) Illustrations of selected areas used for the counting of 5-HTT-ir fibers in the infralimbic (IL) and cingulate (CG) cortices. A grid of 70*70 μm (red) containing three yellow lines with intervals of 17.5 μm was placed over the binary image. (B) Example of the intensity profile of the measurements from one square, each peak represents a detected fiber. [file Image1.TIF]
